# PICK1 inhibits the malignancy of nasopharyngeal carcinoma and serves as a novel prognostic marker

**DOI:** 10.1038/s41419-024-06687-6

**Published:** 2024-04-25

**Authors:** Xiaomin Ou, Yingzi Zhang, Yiqing Xu, Yi Liu, Wenzhi Tu, Chaosu Hu, Yong Liu

**Affiliations:** 1 Department of Radiation Oncology, Fudan University Shanghai Cancer Center, Shanghai, 200032 China; 2 Department of Oncology, Shanghai Medical College, Fudan University, Shanghai, 200032 China; 3 Shanghai Key Laboratory of Radiation Oncology, Shanghai, 200032 China; 4 Department of Radiation Oncology, Shanghai General Hospital, Shanghai Jiao Tong University School of Medicine, Shanghai, 201620 China

**Keywords:** Head and neck cancer, Tumour biomarkers, Tumour-suppressor proteins, Metastasis

## Abstract

Although many important advances have been made in the treatment of nasopharyngeal carcinoma (NPC) in recent years, local recurrence and distant metastasis remain the main factors affecting NPC prognosis. Biomarkers for predicting the prognosis of NPC need to be urgently identified. Here, we used whole-exon sequencing (WES) to determine whether PICK1 mutations are associated with the prognosis of NPC. Functionally, PICK1 inhibits the proliferation and metastasis of NPC cells both in vivo and in vitro. Mechanistically, PICK1 inhibited the expression of proteins related to the Wnt/β-catenin signaling pathway. PICK1 restrained the nuclear accumulation of β-catenin and accelerated the degradation of β-catenin through the ubiquitin-proteasome pathway. The reduced PICK1 levels were significantly associated with poor patient prognosis. Hence, our study findings reveal the mechanism by which PICK1 inactivates the Wnt/β-catenin signaling pathway, thereby inhibiting the progression of NPC. They support PICK1 as a potential tumor suppressor and prognostic marker for NPC.

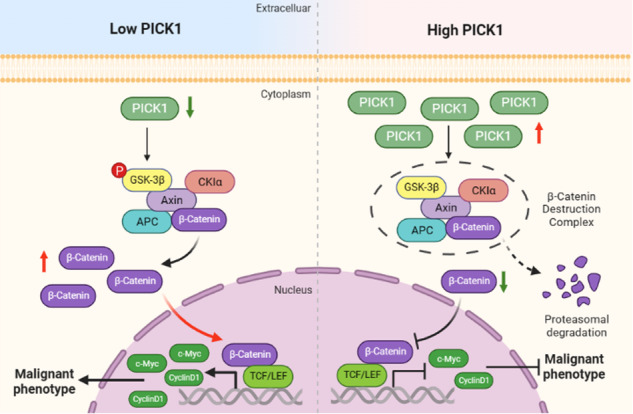

## Introduction

Nasopharyngeal cancer (NPC) is a common malignant epithelial carcinoma found in the head and neck region. NPC has an unbalanced global geographical distribution and is relatively prevalent in South China, Southeast Asia, and North Africa [1]. Currently, it is believed that the occurrence of NPC is ascribed to interactions between environmental factors, genetic predisposition, and Epstein–Barr virus infection [2]. While advances in chemotherapy and intensity-modulated radiotherapy have recently improved the local control, clinical prognosis for NPC patients remains poor due to local recurrence and distant metastasis [3, 4]. Hence, to improve clinical outcomes, it is imperative to elucidate the intrinsic molecular mechanisms involved in the growth and metastasis of NPC.

The Wnt/β-catenin signaling pathway plays a significant role in a variety of physiological processes, such as cell differentiation, proliferation, apoptosis, migration, and cell polarity [5]. Recent studies revealed that this pathway is overactivated in many cancers in humans [6], especially NPC [7, 8]. β-catenin is the core component of this canonical signaling pathway [9]. β-catenin undergoes phosphorylation by glycogen synthase kinase-3β (GSK-3β) in the absence of Wnt, resulting in β-catenin degradation [10]. Upon stimulation by Wnt ligands, β-catenin is stabilized and translocated into the nucleus to stimulate the transcription of Wnt downstream genes [10]. However, there is no evidence demonstrating the connection between PICK1 and Wnt/β-catenin signal alteration in NPC.

Protein interacting with PRKCA 1 (PICK1) has PDZ (PSD-95/Dlg/ZO-1) domain and BAR (Bin/Amphiphysin/Rvs) domain-containing protein, and was first identified by a yeast two-hybrid system based on its interaction with protein kinase C alpha [11]. PICK1 acts as an adapter that interacts with a series of membrane proteins, mediates their subcellular localization and transportation, and is involved in several diseases [12]. Recent studies have highlighted that PICK1 plays a role as a tumor suppressor. Several investigations have demonstrated its ability to inhibit tumor growth and metastasis in various cancers, such as astrocytic cancer [13], prostate carcinoma [14], breast cancer [15], and gastric cancer [16]. Nevertheless, the function and mechanism of PICK1 in the development and progression of NPC have not yet been explored.

In our study, we first performed whole-exome sequencing (WES) of NPC tumors and preliminarily found that PICK1 mutations occurred only in samples obtained from the distant metastasis group. Therefore, we evaluated the role and molecular mechanism of PICK1 in the malignant phenotype of NPC in vitro and in vivo, and analyzed its correlation with clinical prognosis.

## Results

### Somatic mutations in NPC

**Fig. 1:**
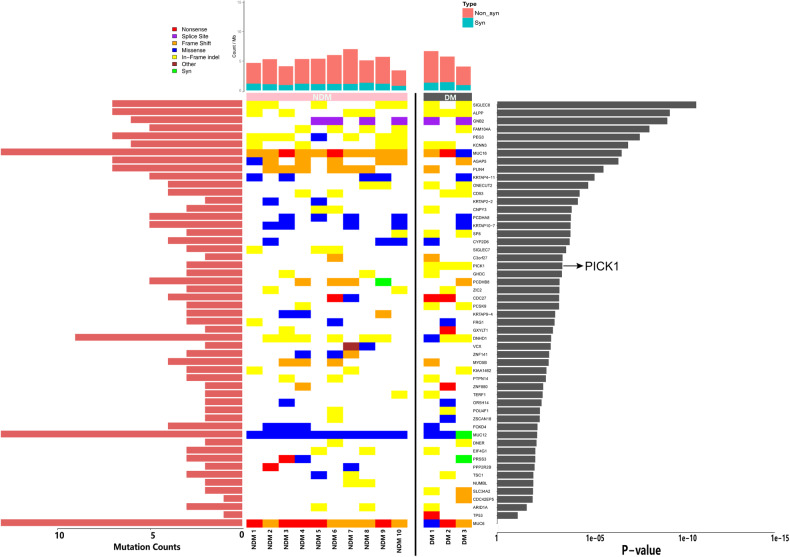
Specific mutated genes between group NDM and group DM. Landscape of somatic mutation profiles in 13 NPC samples. The top panel shows the number of variants in each sample. The mutation types are indicated by diverse colors in the top left panel. The mutation frequency of each gene in all samples is shown as a bar plot in the left panel.

The discovery of this mutation facilitates prognostication and therapeutic planning by illuminating the underlying mechanisms of NPCs. We performed whole-exome sequencing of 13 NPC patients to determine their mutational profiles. Supplementary Table S1 provides an overview of the clinicopathological data of 13 patients with NPC. Somatic mutations were filtered against the criteria used to screen for candidate mutant genes. Figure 1 shows the most mutated genes in all tumor samples sorted by mutational frequency. Next, we counted the number of variants in each gene in the NDM (no distant metastasis) and DM (distant metastasis) groups. Interestingly, PICK1 occurred In-frame indel mutation in all samples from the DM group (Fig. 1). Pancancer prognostic analysis identified in-frame indels as the most important cancer variants affecting patients’ survival [17]. Recent studies have reported that in-frame indel mutations of the tumor suppressor gene AT-rich interactive domain 1 A (ARID1A) in gynecological cancers had substantial impact on cellular proliferation and protein stability [18]. Therefore, we investigated the relationship between PICK1 and NPC progression.

### PICK1 suppresses migration and invasion of NPC cells in vitro

**Fig. 2:**
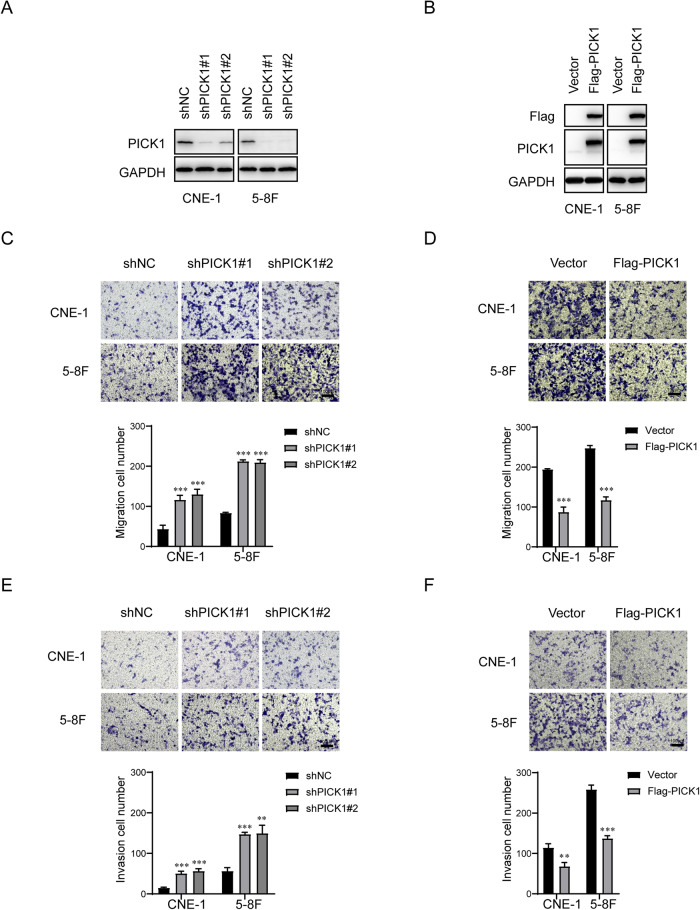
PICK1 suppresses migration and invasion of NPC cells in vitro. **A** PICK1 protein expression in CNE-1 and 5–8 F cells transfected with shNC, shPICK1#1, or shPICK1#2 was examined using western blotting. GAPDH was used as a loading control. **B** Following vector or Flag-PICK1 transfection, PICK1 protein expression in CNE-1 and 5–8 F cells was examined by western blotting. GAPDH was used as a loading control. **C**–**F** Representative pictures and quantification of the impacts of PICK1 knockdown or overexpression on the migratory and invasive capacities of CNE-1 and 5–8 F cells, as assessed by Transwell migration (**C** and **D**) and invasion (**E** and **F**) assays. Each experiment was conducted at least three times. Data are displayed as the mean ± SD. ***p < 0.001, **p < 0.01.

To study the role of PICK1 in NPC, we stably silenced PICK1 using shRNA and exogenously overexpressed PICK1 in CNE-1 and 5–8 F cell lines. Western blotting was used to confirm that PICK1 was effectively overexpressed or knocked down in these NPC cell lines (Fig. 2A, B). We used transwell migration and invasion assays to determine if PICK1 has an impact on NPC cells’ capacity for migration and invasion. As shown in Fig. 2C, transwell migration assays revealed that cells transfected with shPICK1 migrated faster than shNC cells. Transwell invasion assays demonstrated that PICK1 downregulation promoted the invasiveness of CNE-1 and 5–8 F cells (Fig. 2E); As shown in Fig. 2D, PICK1 overexpression significantly suppressed cell migration. Similar results were obtained using transwell invasion assays. The number of invasive cells was lower among PICK1 overexpressing cells (Fig. 2F). These data demonstrate that PICK1 suppresses the migratory and invasive abilities of NPC cells in vitro.

### PICK1 suppresses lung metastasis of NPC cells in vivo

**Fig. 3:**
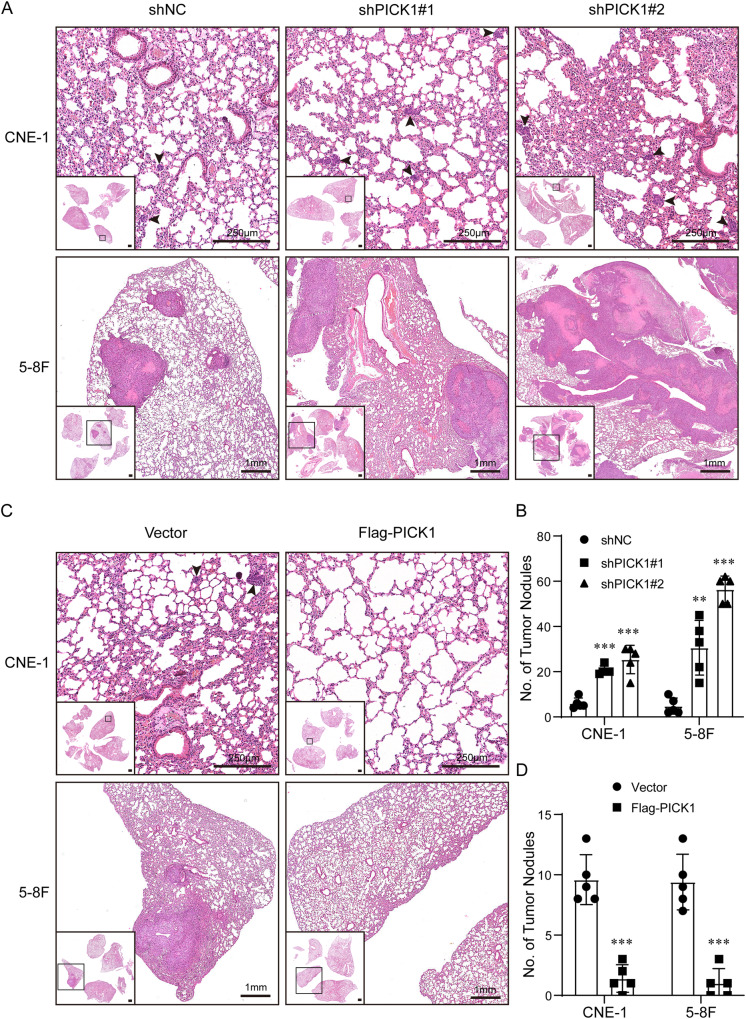
PICK1 suppresses lung metastasis of NPC cells in vivo. **A**, **B** PICK1 stable knockdown and negative control (NC) NPC cells were injected into the tail vein of Nude BALB/c mice (*n* = 5 per group). The mice were euthanized 8 weeks after injection. Representative pictures of lung metastases are shown, and the foci of lung metastasis were evaluated. **C**, **D** NPC cells stably overexpressing PICK1 or vector were intravenously delivered into nude BALB/c mice via the tail vein. The mice were euthanized 8 weeks following injection. Representative images of lung metastasis are shown, and the foci of lung metastases were evaluated.

To further study the function of PICK1 in NPC metastasis in vivo, we created a lung metastasis model through injecting NPC cells with stable knockdown or overexpression of PICK1 into the tail veins of nude mice. Histological analysis of lungs confirmed the presence of metastatic lesions. The number of nodules spread throughout the pulmonary region was noticeably increased in the PICK1 knockdown group compared to the control group (Fig. 3A, B). The lungs of the PICK1-overexpressing group had fewer metastatic nodules than the vector group (Fig. 3C, D). According to these findings, PICK1 significantly reduces NPC metastasis in vivo.

### PICK1 inhibited proliferation of NPC cells in vitro

**Fig. 4:**
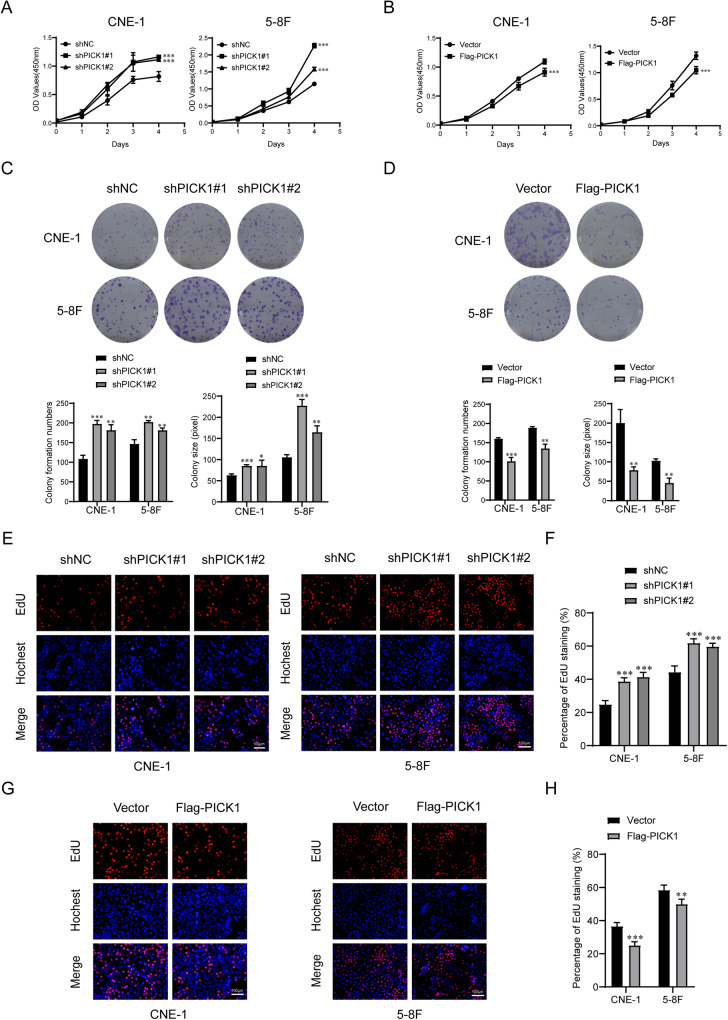
PICK1 inhibits proliferation of NPC cells in vitro. **A**, **B** The viability of the indicated cells was examined using the CCK-8 assay. **C**–**H** The indicated cell proliferation was determined via colony formation assay (**C**, **D**) and EdU Incorporation Assay (**E**–**H**). Representative images and quantitative analyses are shown. The data are displayed as the means ± SD of three independent tests. ***p < 0.001, **p < 0.01, *p < 0.05.

To determine the effect of PICK1 on cell viability and proliferation, we performed CCK-8, colony formation, and EdU assays. These results demonstrated that PICK1 depletion increased cell growth (Fig. 4A), number and size of colonies formed by NPC cells (Fig. 4C), and percentage of EdU-positive cells (Fig. 4E, F) compared to negative control cells. In contrast, CNE-1 and 5–8 F cells with ectopic PICK1 showed cell growth inhibition (Fig. 4B), fewer and smaller colonies (Fig. 4D), and a reduced percentage of EdU-positive cells (Fig. 4G, H). Taken together, these data reveal that inhibition NPC cell proliferation by PICK1 in NPC.

### PICK1 inhibits tumor growth of NPC cells in vivo

**Fig. 5:**
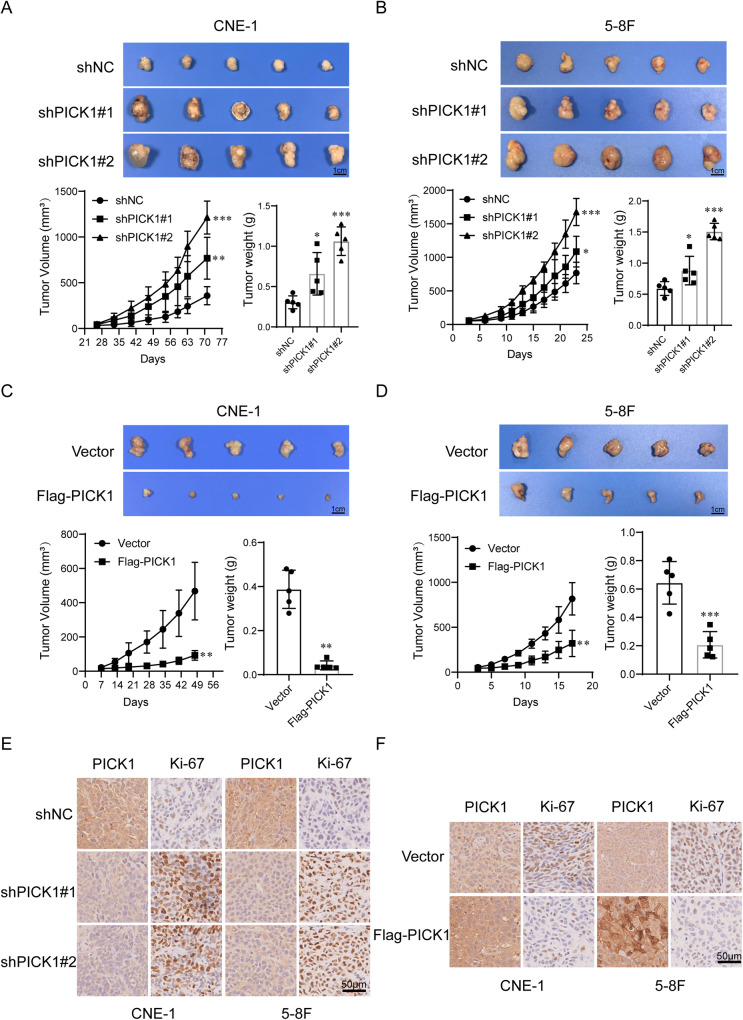
PICK1 inhibits tumor growth of NPC cells in vivo. **A**, **B** Representative images of xenograft tumors, growth curves of tumor volumes, and tumor weights in each group after subcutaneous injections of PICK1 Knockdown or NC in CNE-1 and 5–8 F cells. **C**, **D** Representative images of xenograft tumors, growth curves of tumor volumes, and tumor weights in nude mice inoculated with CNE-1 and 5–8 F cells stably overexpressing PICK1 or the vector. **E**, **F** Immunohistochemistry showing the expression of PICK1 and Ki-67 in mouse tumor tissues. Data are displayed as the mean ± SD. ^***^p < 0.001, ^**^p < 0.01, ^*^p < 0.05.

Next, to study the tumor growth-related effects of PICK1 in vivo, we subcutaneously injected NPC cells into nude mice. Consistent with the in vitro results, PICK1 knockdown stimulated the growth of xenografts compared to controls (Fig. 5A, B), whereas xenografts overexpressing PICK1 exhibited slower growth and decreased tumor weight compared to control xenografts (Fig. 5C, D). The universal proliferation biomarker Ki-67 was used for IHC to determine proliferation. Our findings indicated that the xenograft tumor tissues produced by PICK1-knockdown cells exhibited a considerably elevated Ki-67-staining score, compared to that of the control cells (Fig. 5E). Conversely, xenograft tumor tissues established using PICK1-overexpressing cells showed the opposite effect (Fig. 5F). These observations suggest that the in vivo suppression of NPC cell tumor formation by PICK1 may be a result of cell proliferation inhibition.

### PICK1 inhibited the Wnt/β-catenin signaling pathway

These findings prompted us to investigate the underlying molecular mechanism through which PICK1 inhibits NPC progression. We conducted pathway analysis using GO and KEGG with the mutated genes in NPCs. The most significantly enriched GO and KEGG terms according to gene count and *p*-value are shown in Supplementary Fig. S1A, B, respectively. Regarding the biological processes of GO, the genes were significantly enriched in the Wnt signaling pathway. Additionally, the Wnt signaling pathway was significantly enriched in KEGG.

**Fig. 6:**
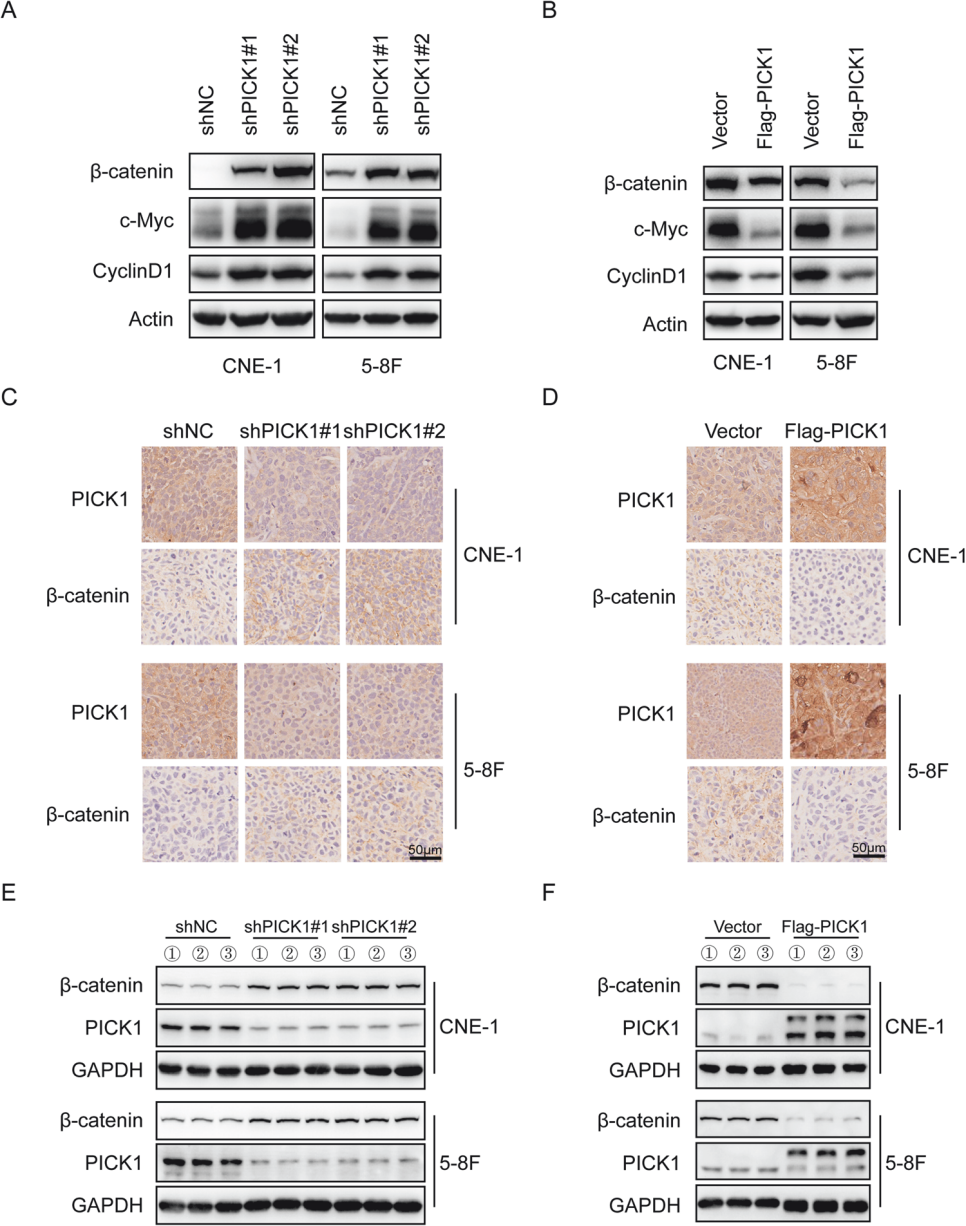
PICK1 inhibits the Wnt/β-catenin signaling pathway. **A**, **B** Western blotting assay was used to examine the expression levels of β-catenin, c-Myc, and cyclinD1 in NPC cells after PICK1 knockdown or overexpression. Actin served as the loading control. **C**, **D** Immunohistochemistry analysis showing the expression of PICK1 and β-catenin in mouse tumor tissues. **E**, **F** PICK1 and β-catenin protein expression in mouse tumor tissues was examined by western blotting.

Therefore, we set out to investigate the potential function of the Wnt/β-catenin pathway in PICK1 inhibiting malignant phenotype in NPC cells. Interestingly, we discovered that the depletion of PICK1 dramatically increased the protein levels of β-catenin (an indicator of classical Wnt pathways) and its downstream targets c-Myc and CyclinD1 in CNE-1 and 5–8 F cells (Fig. 6A), whereas ectopic PICK1 inhibited the expression of β-catenin, c-Myc and CyclinD1 proteins (Fig. 6B).

Moreover, we assessed PICK1 and β-catenin expression in xenograft samples by immunohistochemistry (IHC) and western blotting. Compared to that in control tumors, lower expression of PICK1 in tumors from knockdown cells had higher levels of β-catenin expression. (Fig. 6C, E). Conversely, PICK1 overexpression resulted in its lower expression β-catenin in tumor tissues (Fig. 6D, F). Together, PICK1 inhibited the malignant phenotype of NPC in vivo by regulating the expression of β-catenin, which is line with our in vitro findings.

### PICK1 accelerated proteolytic degradation of β-catenin

**Fig. 7:**
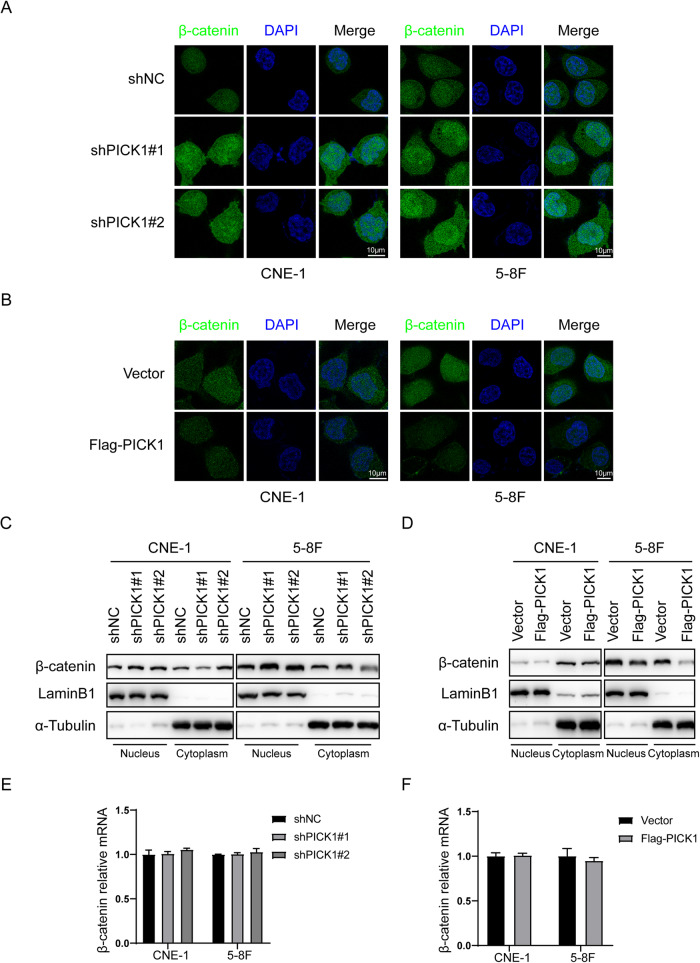
PICK1 promotes β-catenin nuclear accumulation. **A**, **B** Subcellular β-catenin localization in NPC cells was further evaluated by immunofluorescence. **C**, **D** Nuclear and cytoplasmic β-catenin protein levels in NPC cells with overexpressed or silenced PICK1 were measured by western blotting. Internal loading controls used LaminB1 and *α*-Tubulin. **E**, **F** RT-qPCR was used to assess the mRNA levels of β-catenin in the indicated cells.

We looked at the impact of PICK1 on the subcellular distribution of β-catenin. Results from immunofluorescence staining and western blotting revealed that nuclear accumulation of β-catenin in CNE-1 and 5–8 F cells increased following silencing of PICK1 (Fig. 7A, C), whereas nuclear accumulation of β-catenin decreased in NPC cells overexpressing PICK1 (Fig. 7B, D). To figure out whether PICK1 regulates β-catenin at the transcriptional level, we looked at the expression of β-catenin mRNA in cells with PICK1 knockdown or overexpression. These findings revealed that PICK1 did not alter mRNA levels of β-catenin (Fig. 7E, F). Therefore, we postulate that PICK1 may hinder Wnt/β-catenin signaling by affecting the stability of the β-catenin protein.

**Fig. 8:**
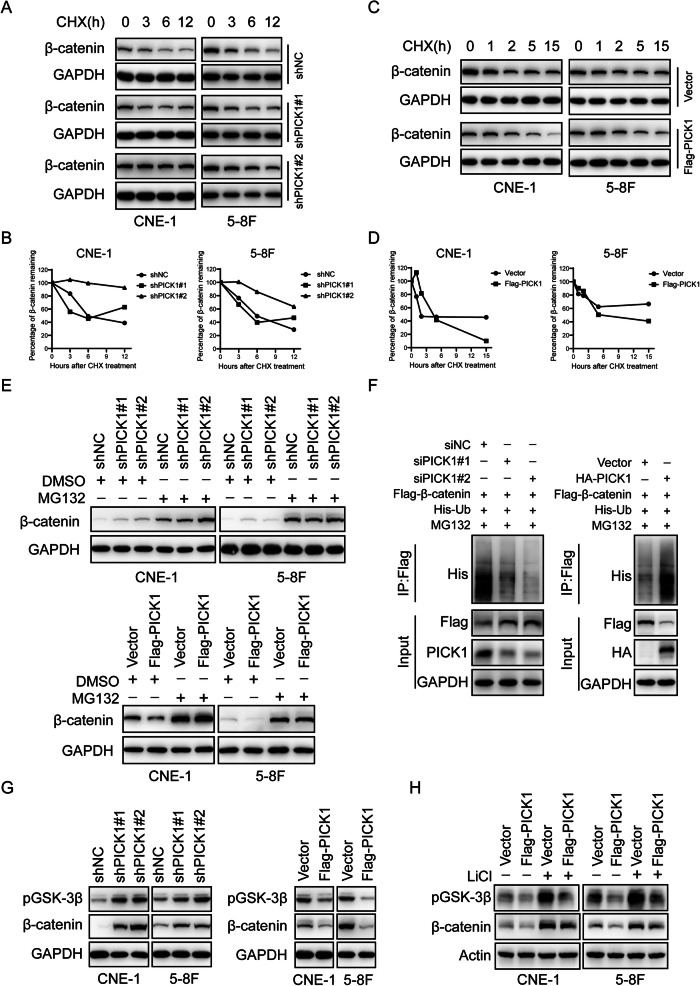
PICK1 accelerates proteolytic degradation of β-catenin. **A**–**E** Western blot analysis of β-catenin protein levels in NPC cells stably knocking down or overexpressing PICK1 treated with CHX (**A**–**D**) and MG132 (**E**). GAPDH acted as the loading control. **F** 293 T cells transfected with the indicated siRNA or plasmids were treated with MG132 (10 μM) for 6 h before collection. Ubiquitylation assays were conducted and the ubiquitylation level of β-catenin was analyzed using an anti-His antibody. **G** Western blot analysis of pGSK-3β and β-catenin expression in NPC cells with stably silenced PICK1 or in which PICK1 was stably overexpressed. GAPDH served as the loading control. **H** Western blot analysis of pGSK-3β and β-catenin levels in NPC cells with vector or Flag-PICK1, followed by treatment with or without LiCl (20 mM) for 24 h. Actin served as the loading control.

To test this hypothesis, we treated NPC cells with cycloheximide (CHX) to prevent the synthesis of β-catenin and measured its levels to determine the degradation rate of β-catenin. Western blotting results showed β-catenin protein levels dropped much faster in the control CNE-1 and 5–8 F cells than in the cells with PICK1 knockdown (Fig. 8A, B, Supplementary Fig. S2A), while overexpression of PICK1 significantly accelerated the degradation of β-catenin, resulting in a significant reduction in its expression (Fig. 8C, D, Supplementary Fig. S2B). Next, we examined the expression of β-catenin in NPC cells treated with MG132, an 26 S protostome inhibitor, and observed that MG132 reversed the downregulation of β-catenin protein induced by PICK1 (Fig. 8E). Additionally, ubiquitination assay indicated that ubiquitination of β-catenin was weakened in the absence of PICK1, and on the contrary, ubiquitination of β-catenin was raised in the presence of PICK1 (Fig. 8F), which explained the accelerating degradation of β-catenin protein.

It is well known that GSK-3β-mediated phosphorylation and subsequent β-TrCP1 binding for targeted ubiquitylation and degradation of β-catenin regulate its stability in the canonical Wnt pathway [19]. We hypothesized that PICK1 may protect β-catenin from GSK-3β-mediated protein degradation. We observed that the depletion of PICK1 in CNE-1 and 5–8 F cells increased the expression of GSK-3β Ser9 phosphorylation and β-catenin. By contrast, PICK1 overexpression decreased GSK-3β Ser9 phosphorylation and β-catenin expression (Fig. 8G). As an inhibitor of GSK-3β, LiCl can directly activate β-catenin, thus activating the Wnt/β-catenin pathway. The results showed that LiCl significantly upregulated GSK-3β Ser9 phosphorylation and β-catenin protein, and it significantly reversed PICK1-induced β-catenin downregulation, confirming the validity of LiCl and demonstrating that LiCl could reverse PICK1-induced inhibition of the Wnt/β-catenin pathway (Fig. 8H).

Based on our findings, we suggest that depletion of PICK1 inhibits GSK-3β activity by promoting GSK-3β phosphorylation at Ser9 to stabilize β-catenin through the ubiquitin-proteasome pathway, which stimulates Wnt/β-catenin signaling.

### The PICK1 contents were negatively associated with worse prognosis among NPC patients

To further establish the pathological connection between PICK1 and the clinical characteristics of human NPC, we conducted IHC staining for PICK1 in primary NPC samples, and the results showed PICK1 expression at different intensities (Supplementary Fig. S3A). Notably, cancer patients with significantly reduced levels of PICK1 in their tumors experienced shorter overall survival (OS), progression-free survival (PFS), and distant metastasis-free survival (DMFS) than those with higher levels of PICK1 (Supplementary Fig. S3B–D). Additionally, we found a significant correlation between PICK1 expression and the clinical stage (*p* = 0.0016) and T stage (*p* = 0.0199) (Supplementary Table S2). These findings collectively indicate that dysregulation of PICK1 potentially contributes to malignant progression and can be used to predict poor prognosis in patients with NPC.

## Discussion

According to established literature [20, 21], the incidence of local invasion and early metastasis in NPC is notably high. Although intensity-modulated radiotherapy has had revolutionary effects in improving local control, distant metastasis continues to significantly impact treatment failure and patient mortality in NPC [22, 23]. Consequently, the identification of specialized biomarkers for detecting metastasis in high-risk patients with NPC is of immense importance. Such biomarkers could inform the design of novel treatment strategies and provide hope for patients in the future. In the present study, we performed whole-exome sequencing (WES) of 13 NPC samples to obtain comprehensive genomic information. Our findings revealed that PICK1 was mutated in every sample in the group with distant metastases, suggesting that it may play a crucial role in NPC tumor progression. Therefore, we investigated the functional role of PICK1 in NPC.

Our results indicate that the knockdown of PICK1 significantly enhanced cell migration, invasion, and proliferation. Conversely, the overexpression of PICK1 diminished cell invasion and proliferation in NPC cells. Nude mice were used to evaluate the role of PICK1 in vivo. Forced PICK1 expression suppresses tumor growth and lung metastasis in NPC cells, while the depletion of PICK1 promoted tumor growth and lung metastasis. As documented previously, the expression of PICK1 is downregulated in grade IV astrocytic tumor cell lines and is associated with tumor progression [13]. Similarly, in prostate carcinoma cells, PICK1 suppresses both cell invasion and migration and bone metastasis [14]. Additionally, the downregulation of PICK1 promotes the metastasis of breast cancer cells [15]. Other studies have demonstrated that PICK1 expression is significantly reduced in gastric tumor tissues, which has a negative impact on the overall survival of patients with gastric carcinoma [16]. These previously published findings are in agreement with those of the present study.

The underlying PICK1 regulation mechanisms in NPC need to be further investigated. As evidenced by the GO and KEGG enrichment analyses of all mutated genes, the Wnt signaling pathway may be implicated in the downstream mechanisms. It is well documented that abnormalities in the Wnt/β-catenin pathway are widely present in various types of malignancies [24]. The canonical Wnt signaling pathway governs the transcription of numerous genes, with β-catenin acting as the central mediator of this pathway [25]. Prolonged and abnormal activation of the Wnt/β-catenin signaling pathway enable the accumulation of β-catenin and its subsequent entrance into the nucleus, activating downstream TCF/LEF family regulatory factors to perform their corresponding regulatory role [26]. Recent findings suggest that METTL1 can upregulate the WNT/β-catenin signaling pathway, promoting NPC cell chemoresistance to cisplatin and docetaxel and epithelial-mesenchymal transition (EMT) in vitro and in vivo [27]. NUSAP1 potentiates Wnt/β-catenin signaling and facilitates the proliferation and invasion of NPC cells [28]. Our study indicates that PICK1 inhibits the expression of β-catenin, c-Myc, and CyclinD1, which are well-known targets of the Wnt/β-catenin signaling pathway. These results were validated using a mouse xenograft model.

Moreover, it was discovered through our research that overexpression of PICK1 resulted in the reduction of β-catenin protein levels, but not mRNA levels. The observed downregulation of β-catenin induced by PICK1 was reversible by the proteasome inhibitor MG132, indicating that PICK1 promotes the downregulation of β-catenin through a proteasome-dependent pathway. The further Ubiquitination assay elucidated that PICK1 restricted the Wnt/β-catenin signaling pathway by promoting the ubiquitination of β-catenin and subsequently destabilizing its protein. It is noteworthy that the cytoplasmic GSK-3β functions to regulate β-catenin degradation via phosphorylation as part of the WNT signaling pathway, along with APC and Axin [29]. Previous reports suggested that GSK-3β is often inactivated in NPC specimens, resulting in the accumulation of nuclear β-catenin [30, 31]. The down-regulation of GSK-3β causes β-catenin to accumulate in the nucleus, activating downstream effectors such as c-Myc and CyclinD1, which promote cell cycle progression and proliferation [32, 33]. As predicted, our data indicated that PICK1 decreased β-catenin expression by inhibiting Ser9 phosphorylation and promoting GSK-3β activity, ultimately blocking the nuclear accumulation of β-catenin.

The present findings indicate that PICK1 possesses considerable potential to suppress the proliferation and metastasis of NPC cells both in vitro and in vivo. Additionally, we demonstrated the critical role of PICK1 in regulating β-catenin stability by serving as a novel inhibitor of Wnt signaling. Specifically, we discovered that patients with low levels of PICK1 in NPCs exhibited poorer survival outcomes during our analysis of NPC specimens using IHC assays on a tissue microarray.

In summary, our results identified a PICK1 mutation associated with NPC progression. The present data demonstrate that PICK1, a tumor suppressor gene, inhibits the proliferation and metastasis of NPC cells both in vivo and in vitro. Moreover, we have elucidated the function of the PICK1/GSK-3β/β-catenin axis in NPC. Therefore, we indicate that PICK1 is a novel prognostic biomarker and a potential therapeutic target for NPC. It is necessary to acknowledge the limitations of this work. The sample size for WES to determine somatic mutations of NPC patients is relatively limited, which may have affected the generalizability of this work. With regard to this, we are planning to launch a larger cohort size study to make up the limitations.

## Material and methods

### Patients and Samples

The Medical Ethics Committee of Fudan University Shanghai Cancer Center granted approval for this study. Thirteen patients were recruited between 2007 and 2011 and written informed consent was obtained from each participant. All samples were pathologically confirmed as NPC. For this study, distant metastasis was defined as the presence of clinical evidence of a distant disease, as determined by clinical and radiographic findings. The complete clinical characteristics of the patients selected for sequencing are listed in Supplementary Table S1.

### WES/ Bioinformation analysis strategy

Sequencing and bioinformatics analyses of whole exons from NPC specimens were performed in partnership with Shanghai OE Biotech Co., Ltd. High-frequency mutant genes linked to distant metastasis (DM) and non-distant metastasis (NDM) of NPC were screened. GO and KEGG pathway enrichment analysis for the mutated genes of NPC were visualized with the cluster Profiler package in R, and significance was determined based on a *p-*value <0.05.

### Cell lines and culture

Human NPC cell lines, CNE-1 and 5–8 F, were purchased from Central South University (China). These two cell lines were maintained in RPMI-1640 medium (Wisent, Canada) with 10% fetal bovine serum (FBS, Wisent, Canada), 100 μg/mL streptomycin, and 100 U/mL penicillin (NCM Biotech, China). The Cell Bank of the Chinese Academy of Sciences provided the 293 T cells. Dulbecco’s modified Eagle’s medium (DMEM; Wisent, Canada) containing 10% FBS and penicillin-streptomycin was used to cultivate 293 T cells. All cells were cultured in a humidified incubator at 37 °C with 5% CO_2_.

### Plasmids, siRNAs, and reagents

HA-PICK1, His-Ub, and Flag-β-catenin plasmids were provided by OBiO Technology Co., Ltd. (China). The siRNA sequences were as follows, siPICK1#1: CTTGGATTATGACATCGAA. siPICK1#2: CTCCCTGCCTCTATA TCGT. Negative control siRNA was provided by RiboBio (China). MG132 and Lithium chloride (LiCl) were purchased from Sigma (USA).

### Stable cell line construction

The overexpression plasmids were inserted into the pLenti-EF1a-Puro. A specific short hairpin RNA (shRNA) sequence was designed and cloned into the pSLenti-U6-shRNA. The lentiviruses were generated using 293 T cells and packaging plasmids (psPAX2 and pMD2.G). NPC cells were infected with the lentivirus in the existence of polybrene. The cells were gathered for protein expression assays.

### Cell proliferation assay

For cell proliferation, the cells were initially planted in 96-well plates at a density of 2000 cells/well (CNE-1) or 1000 cells/well (5–8 F) and incubated for 0, 1, 2, 3, and 4 days. Then, the cells were subjected to treatment utilizing with Cell Counting Kit-8 (CCK-8; Dojindo, Japan) for 1 h. The absorbance of each well was quantified at 450 nm using an EPOCH 2 microplate reader (BioTek Instruments, Inc., USA). This assay was performed in triplicate.

### Colony formation assay

Cells were planted in 6-well culture plates at a density of 200 cells/well, with three wells per group. After incubation for 9 d at 37 °C, colonies were washed twice with PBS, fixed with methyl alcohol for 15 min and stained with Crystal Violet Staining Solution (Beyotime, China) for 15 min. Visible colonies were counted manually. All experiments were repeated at least thrice.

### EdU Incorporation assay

Cells were seeded in 48-well plates at a density of 1 × 10^4^ cells/well and cultured for 24 h. Proliferating cells were examined using the Cell-Light^TM^ EdU Apollo 567 In Vitro Kit (RiboBio, China) per the manufacturer’s instructions. After being exposed to 50 µM EdU for 2 h, the cells were fixed with 4% paraformaldehyde, permeabilized in 0.5% Triton X-100 and stained with 1 X Apollo^®^ reaction cocktail, with 1 X Hoechst 33342 used to stain cell nuclei for 30 min. A fluorescence microscope (Leica, Germany) was used to count EdU-positive cells in five random fields. All assays were performed independently three times.

### Cell migration and invasion assay

Transwell chambers (Corning, USA) were used to conduct cell migration assays. The Transwell membrane was pre-coated with Matrigel (BD Biosciences, USA) for the cell invasion experiment. The bottom chamber included RPMI-1640 with 10% FBS to serve as a chemoattractant, and the upper chamber contained CNE-1 (overexpression/knock-down: 2/1.5 × 10^5^ for the migration assay; 4/3 × 10^5^ for the invasion assay) and 5–8 F cells (overexpression/knockdown: 1/0.75 × 10^5^ for the migration assay; 2/1.5 × 10^5^ for the invasion assay) in serum-free medium. The non-invading cells on the filters’ upper surface were eliminated using a cotton swab after 24 h of incubation. The lower surface of the filter that had invasive cells adhered to it was washed with PBS, preserved with methanol, and stained with 0.1% crystal violet. Under a microscope, the number of migrating or invading cells was determined by counting cells in three random fields.

### RNA extraction, reverse transcription and Real Time Quantitative PCR

Total RNA was isolated from NPC cells using TRIzol reagent (Invitrogen, USA) following the manufacturer’s instructions. Hifair^®^III 1st Strand cDNA Synthesis SuperMix for qPCR (gDNA digester plus) (Yeasen Biotechnology, China) was utilized to reverse transcribe 1 μg total RNA into cDNA. On a QuantStudio 6 Flex system (Life Technologies, USA), qPCR was carried out using Hieff UNICON^®^ Universal Blue qPCR SYBR Green Master Mix (Yeasen Biotechnology, China). The primers for CTNNB1 were: forward 5’-TGGATTGATTCGAAATCTTGCC-3’ and reverse 5’- GAACAAGCAACTGAACTAGTCG -3’. GAPDH served as a normalization control. The relative expression of target genes was calculated using the 2^−ΔΔCT^ method. Each sample was examined in triplicates.

### Western blotting analysis

The RIPA lysis buffer (Beyotime, China) containing a proteinase and phosphatase inhibitor cocktail (Bimake, USA) was used to isolate the total protein. Proteins were extracted from the tissues using a Column Tissue&Cell Protein Extraction Kit (Epizyme, China). The protein concentration was measured with the Enhanced BCA Protein Assay Kit (Beyotime, China). Aliquots of protein were subsequently separated on 10%–12.5% sodium dodecyl sulfate polyacrylamide gel electrophoresis (SDS-PAGE) and transferred onto PVDF membranes (Millipore, USA). After blocking with 5% skim milk, the membranes were treated with primary antibodies and the corresponding secondary antibodies. Finally, protein bands were detected utilizing enhanced chemiluminescence (ECL, Millipore, USA) and visualized using an imaging system (Tanon, China). Primary antibodies were as follows: PICK1 (1:1,000, CST, 85325), GAPDH (1:3,000, CST, 5174), Flag (1:5,000, Sigma, F1804), β-catenin (1:10,000, Abcam, ab32572), c-Myc (1:1,000, CST, 5605), CyclinD1 (1:10,000, Abcam, ab134175), Actin (1:1,000, CST, 4970), Lamin B1 (1:10,000, Proteintech, 66095-1-Ig), a-Tubulin (1:5,000, Sigma, T6074), His (1:1,000, CST, 12698), HA (1:5000, Sigma, H6908), Phospho-GSK-3β (Ser9) (1:1,000, CST, 5558).

### Immunofluorescence staining

CNE-1 and 5–8 F cells were grown on confocal dishes to 90% confluence, after which they were fixed for 30 min with 4% paraformaldehyde, permeabilized for 15 min with 0.1% Triton X-100, and blocked for 30 min with 1% bovine serum albumin (BSA). Subsequently, cells were incubated with the primary antibody (β-catenin, Abcam, ab32572, 1:250) overnight at 4 °C. The next day, the cells were stained for 1 h with an Alexa Fluor 488-conjugated secondary antibody (1:1000, Abcam) at room temperature. After washing with PBS, the nuclei were counterstained with DAPI (Sigma, USA) at room temperature for 10 min. Images were captured and recorded using a Leica SP8 laser-scanning confocal microscope (Leica, Germany).

### In vivo mouse models

Jiangsu GemPharmatech Co. Ltd. (China) provided us with specific pathogen-free (SPF) BALB/c nude male mice (5–6 weeks), the mice were randomly divided. The Ethics Committee for Animal Research at Shanghai General Hospital gave its approval to all animal trials. For tumor xenograft model, CNE-1 cells (overexpression/knockdown:5/2.5 × 10^6^), or 5–8 F cells (overexpression/knockdown:5/2 × 10^6^) stably overexpressing or knocking down PICK1 were subcutaneously inoculated into the right flank of the mice. The mice were monitored for palpable tumor formation at regular intervals, and the tumors were measured, weighed, and photographed. Subcutaneous tumors were obtained for protein and histological analyses using western blotting and immunohistochemistry, respectively. For the tumor lung metastasis model, CNE-1 cells or 5–8 F cells with PICK1 stably upregulated and downregulated were injected into mice via the tail vein. The mice were sacrificed after 8 weeks, and all lung tissues were collected, fixed, sectioned, and stained with hematoxylin and eosin (H&E). Five mice were used for each experimental group. No animals were excluded from the analysis.

### Nuclear and cytoplasmic protein extraction

According to the manufacturer’s instructions, a nuclear and Cytoplasmic Protein Extraction Kit (Beyotime, China) was used to segregate the cytoplasmic and nuclear proteins. Lamin B1 and a-Tubulin were served as references for nuclear and cytoplasmic proteins, respectively.

### Cycloheximide chase assay

CNE-1 and 5–8 F cells stably overexpressing PICK1 or knocking down PICK1 were planted in 6 cm cell culture plates and cultured for 24 h. In NPC cells, 100 μg/mL cycloheximide (CHX; Sigma) was included to the culture media too prevent de novo protein synthesis. At the designated times, cells were harvested, lysed, and submitted to western blotting analysis.

### Hematoxylin and Eosin (H&E) and Immunohistochemistry (IHC) staining

Samples of mice lung tissue and tumor tissue were fixed at least 24 h in 4% paraformaldehyde solution. The tissues were sliced into 6 mm thick sections after gradient dehydration and paraffin embedding, deparaffinized in xylene, and rehydrated using a gradient of ethanol solutions with progressively lower concentrations. The sections were utilized for H&E staining to assess alterations in tissue structure and tumor cell metastasis. Primary antibodies against PICK1 (1:100, Proteintech, 10983-2-AP), Ki-67 (1:12,000, Abcam, ab15580), and β-catenin (1:50, Abcam, ab32572) were used to conduct IHC analysis of the tissue samples. Staining results were evaluated and photographed using a microscope (Leica, Germany).

### Ubiquitination assay

Cells were transfected for 48 h with plasmids expressing His-Ub and Flag-β-catenin (together with siRNA or HA-PICK1 plasmids). Cells were incubated with 10 µM MG132 for 6 h before collection. Cells were washed with cold PBS and lysed in 500 µL lysis buffer (Absin, China). Before lysates were detected by SDS-PAGE, they were immunoprecipitated overnight with 1 µg anti-Flag antibody at 4 °C and washed 3 times with cold wash buffer. After that, they were conducted to ubiquitination analysis using anti-His antibody by western blotting.

### Human NPC tissue microarray (TMA)

We acquired human NPC TMA from Shanghai Outdo Biotech Co., Ltd. IHC analysis consisted of 99 cases after excluding factors such as exfoliation and insufficient cancerous tissue. TMA encompasses comprehensive clinicopathological features, including age, sex, TNM stage, and location, along with prognostic information. PICK1 antibody was used at a dilution of 1:400. The mean score was obtained by combining the assessments provided by the two independent pathologists. Tumor cell proportions were evaluated using the following scoring system: 0, no positive tumor cells, 1 for 1–25% positive tumor cells, 2 for 26–50% positive tumor cells, 3 for 51–75% positive tumor cells; and 4, >75% positive tumor cells. The staining intensity was categorized into four grades:0, no staining, 1 representing weak staining, 2 indicating moderate staining; and 3, strong staining. The staining index (SI) was determined by multiplying the staining intensity score with the proportion of positive tumor cells. Samples classed as demonstrating high expression had an SI >6, while those classified as displaying low expression had an SI of 6 or less.

### Statistical analyses

Quantitative data were presented as the mean ± SD from a minimum of three independent experiments. Using either a paired Student’s *t*-test or an unpaired Student’s *t*-test with two tails, differences between the two groups were examined. The statistical significance of the association between PICK1 protein levels and clinicopathological characteristics was evaluated using the chi-square test. Survival curves were estimated with the Kaplan-Meier method and subsequently compared using log-rank tests. *P*-value was set at *p* < 0.05 indicates significance. All analyses were performed using the GraphPad Prism 8 software (GraphPad Software Inc.).

### Supplementary information

Supplementary material

Original Data File

## Data Availability

All data generated or analyzed during this study are included in this published article and its supplementary information files.
