# Supplementary material for: PICK1 inhibits the malignancy of nasopharyngeal carcinoma and serves as a novel prognostic marker
Source: Cell Death Dis. 2024 Apr 25;15(4):294. doi: 10.1038/s41419-024-06687-6 (PMC11045752; doi:10.1038/s41419-024-06687-6)
Supplement: Supplementary file 1 — Supplementary material [file 41419_2024_6687_MOESM1_ESM.docx]

**Supplementary figures**


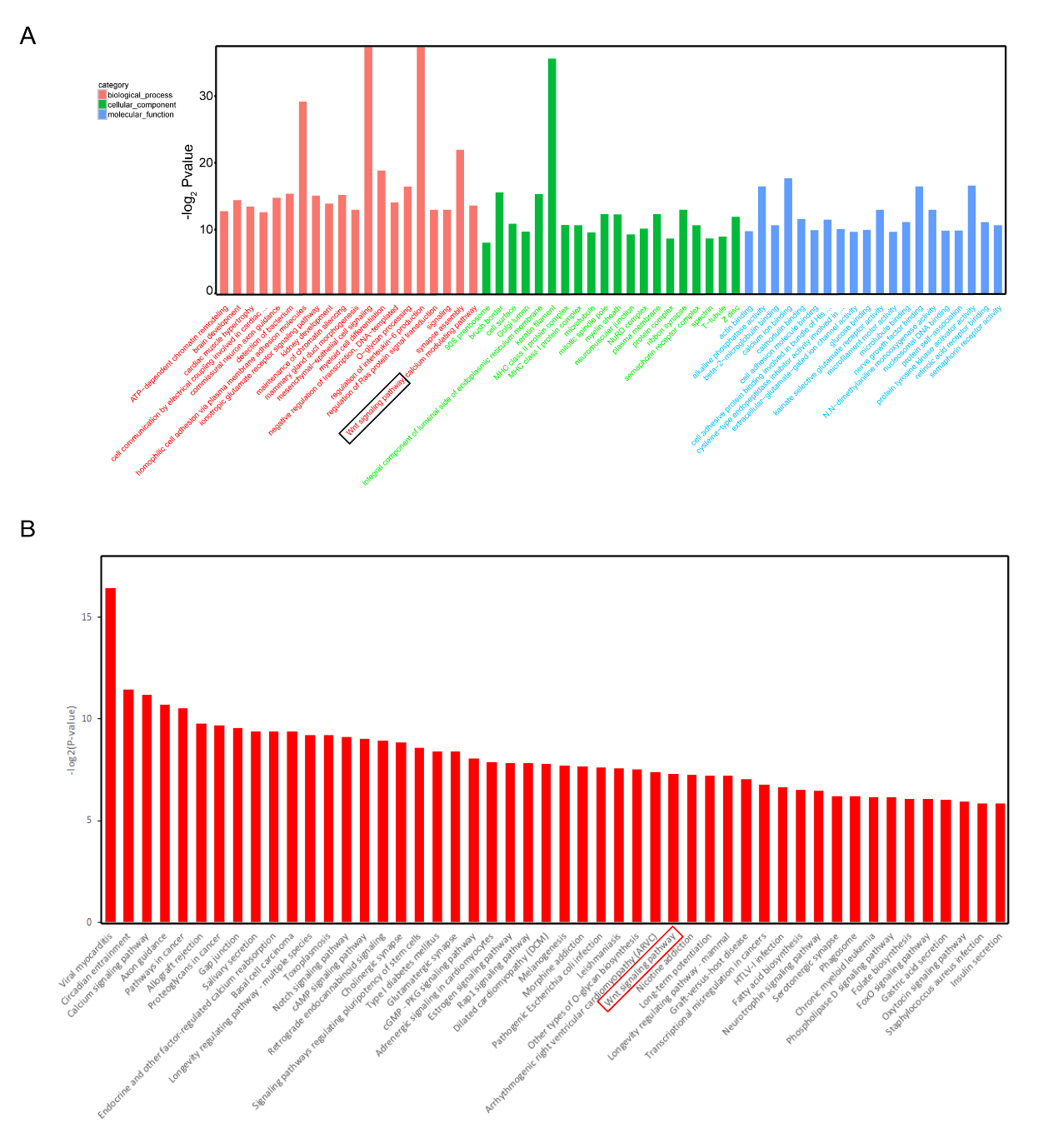


**Figure S1.** **PICK1 is related to Wnt signaling pathway.**

**A, B** Enrichment of mutated genes by GO and KEGG analyses. (A) GO enrichment analysis. (B) KEGG enrichment analysis.


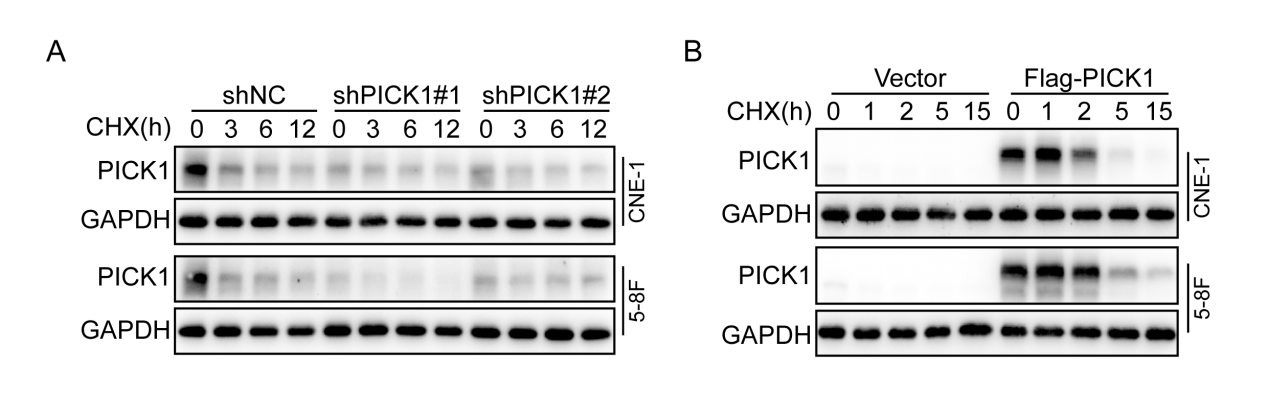


**Figure S2. The expression of PICK1 in NPC cells in CHX chase assay.**

**A, B** PICK1 protein expression in CNE-1 and 5-8F cells stably knocking down or overexpressing PICK1 treated with CHX was examined using western blotting. GAPDH was used as a loading control.


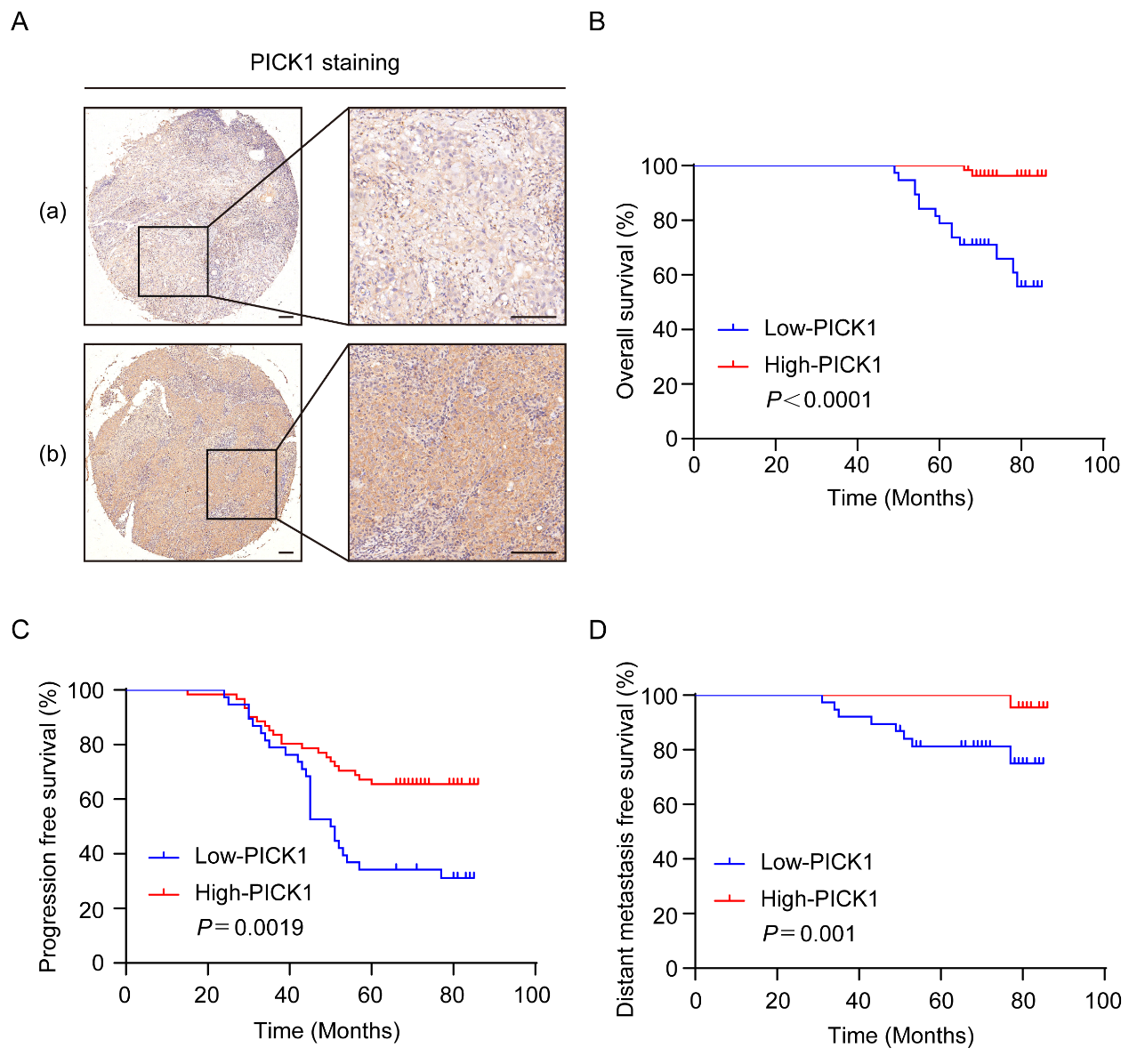


**Figure S3. The PICK1 contents are negatively associated with worse prognosis among NPC patients.**

**A** Representative IHC staining of weak (a) and strong (b) PICK1 expression in a tissue microarray of patients with NPC. Scale bar: 100 μm.

**B-D** Kaplan-Meier survival curves of overall survival (B), progression-free survival (C), and distant metastasis-free survival (D) in patients with high and low PICK1 expression. The log-rank test was utilized to analyze the data.

**Supplementary tables**

Table S1. **Clinical Characteristics of 13 NPC Patients.**

| Characteristic | NPC-NDM^a^  ( n = 10 ) | NPC-DM^b^  ( n = 3 ) | *P value* | All NPC  ( n = 13 ) |
| --- | --- | --- | --- | --- |
| Age |  |  | 0.1005 |  |
| Media (rang) | 43 (25 - 62) | 46 (43 - 49) |  | 44 (25 - 62) |
| ＜40 | 4 | 0 |  | 4 |
| 41-50 | 2 | 3 |  | 5 |
| 51-60 | 3 | 0 |  | 3 |
| ＞60 | 1 | 0 |  | 1 |
| Gender |  |  | ＞0.9999 |  |
| Male | 8 | 3 |  | 11 |
| Female | 2 | 0 |  | 2 |
| Clinical stage |  |  | 0.1644 |  |
| II | 2 | 1 |  | 3 |
| III | 2 | 2 |  | 4 |
| IV | 6 | 0 |  | 6 |
| Smoking status |  |  | 0.5594 |  |
| Smoker | 4 | 2 |  | 6 |
| Non-smoker | 6 | 1 |  | 7 |

a: NDM, no distant metastasis after treatment; b: DM, distant metastasis after treatment.

Table S2. **Correlations between PICK1 expression and clinical characteristics in NPC patients.**

| Clinicopathologic parameters | Case  (n = 99) | PICK1 expression | | *P value* |
| --- | --- | --- | --- | --- |
|  |  | Low | High |  |
| Total | 99 | 38 | 61 |  |
| Gender |  |  |  | 0.1097 |
| Male | 71 | 31 | 40 |  |
| Female | 28 | 7 | 21 |  |
| Age |  |  |  | 0.6803 |
| ≥ 50 | 42 | 15 | 27 |  |
| < 50 | 57 | 23 | 34 |  |
| Clinical stage |  |  |  | 0.0016 |
| I-III | 83 | 26 | 57 |  |
| IV | 16 | 12 | 4 |  |
| T stage |  |  |  | 0.0199 |
| T1-3 | 88 | 30 | 58 |  |
| T4 | 11 | 8 | 3 |  |
| Lymph node metastasis |  |  |  | 0.0700 |
| N0-2 | 94 | 34 | 60 |  |
| N3 | 5 | 4 | 1 |  |
| Distant metastasis |  |  |  | 0.3838 |
| M0 | 98 | 37 | 61 |  |
| M1 | 1 | 1 | 0 |  |
