# Supplementary figures and images for: PICK1 inhibits the malignancy of nasopharyngeal carcinoma and serves as a novel prognostic marker
Source: Cell Death Dis. 2024 Apr 25;15(4):294. doi: 10.1038/s41419-024-06687-6 (PMC11045752; doi:10.1038/s41419-024-06687-6)

Figure 2AB

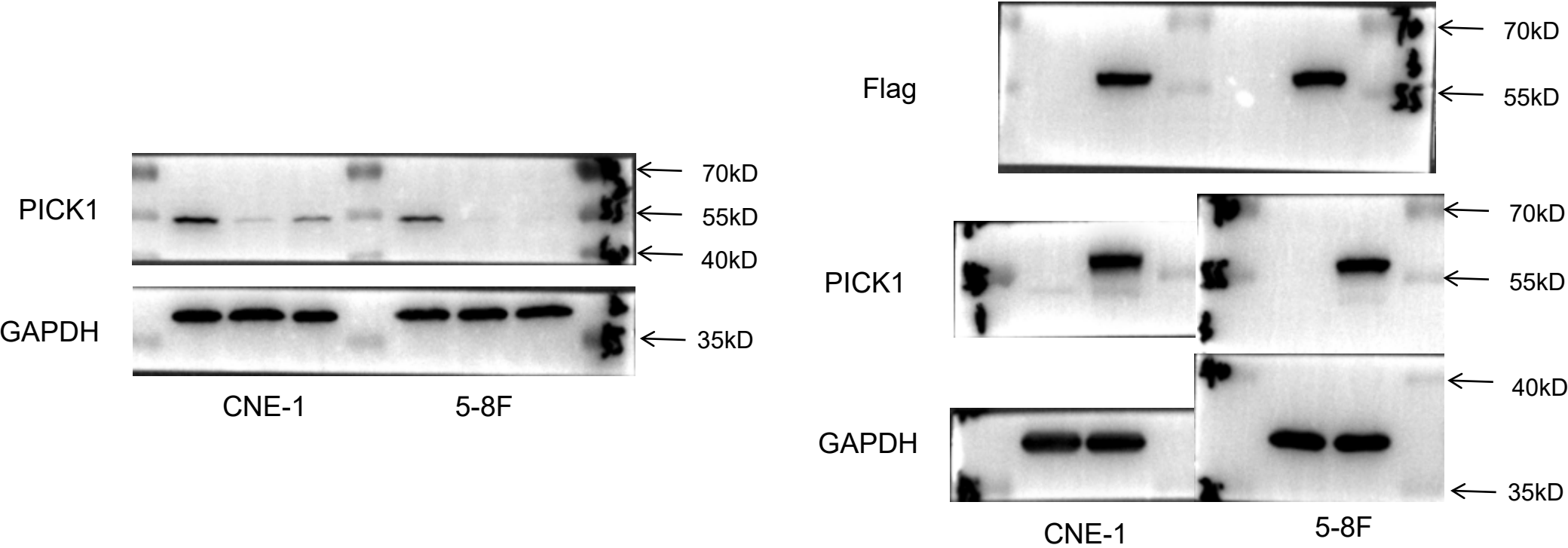

Figure 6AB

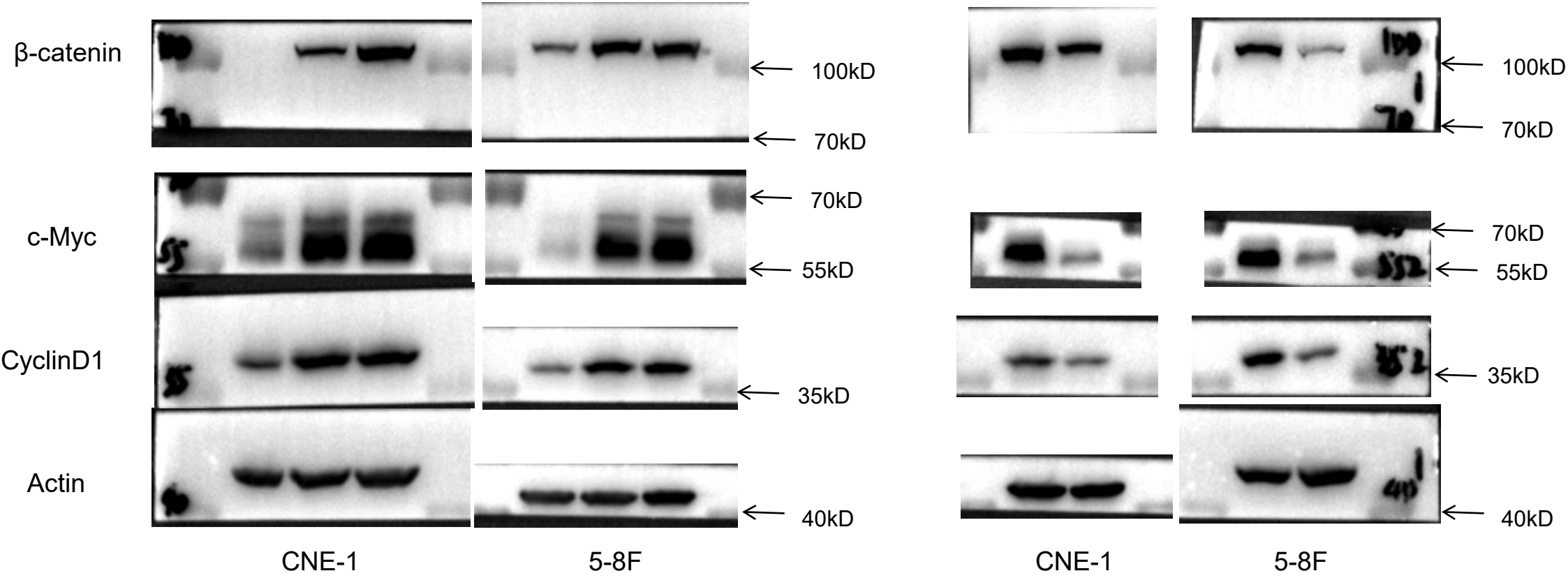

Figure 6EF

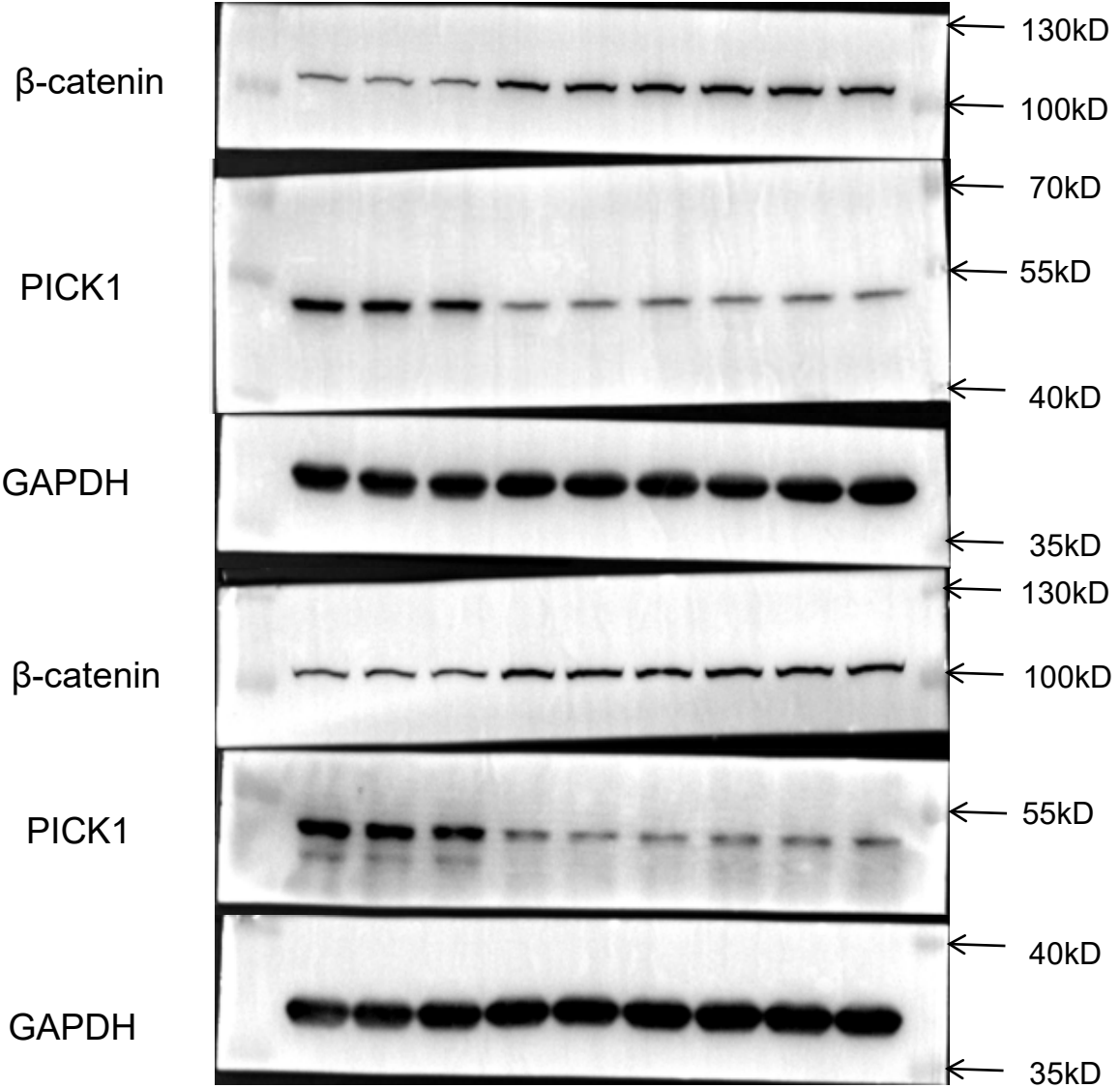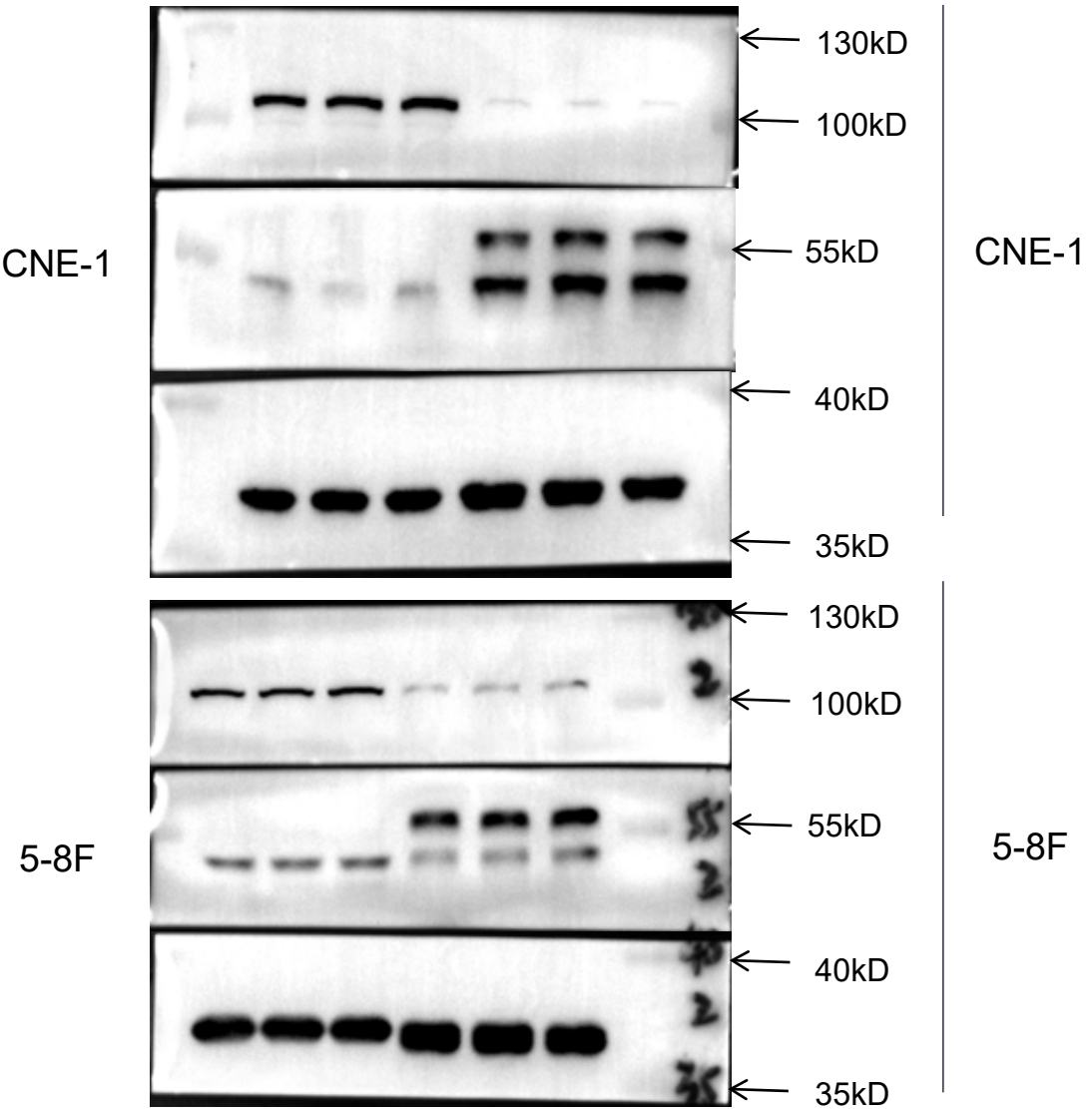

Figure 7CD

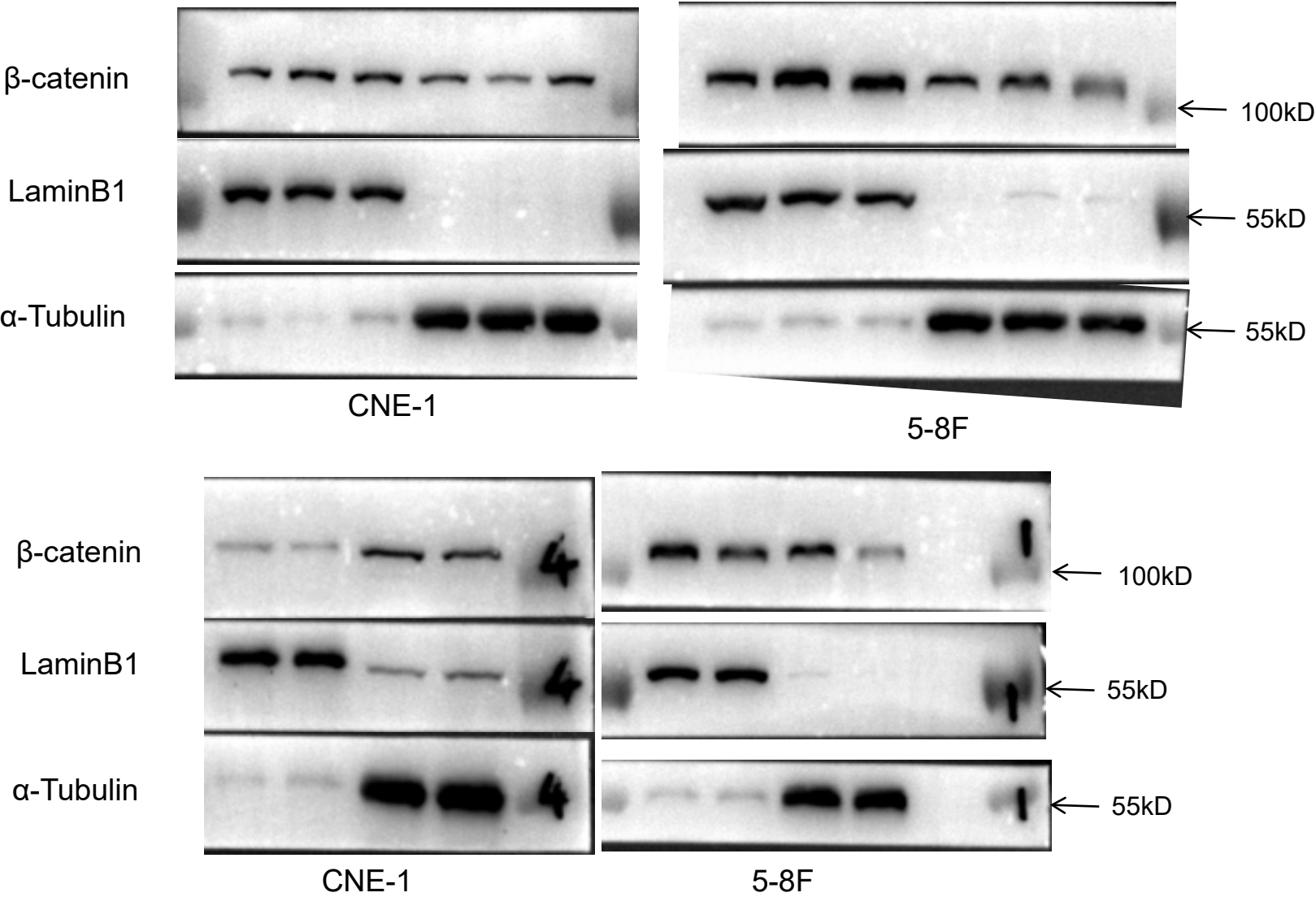

Figure 8AC

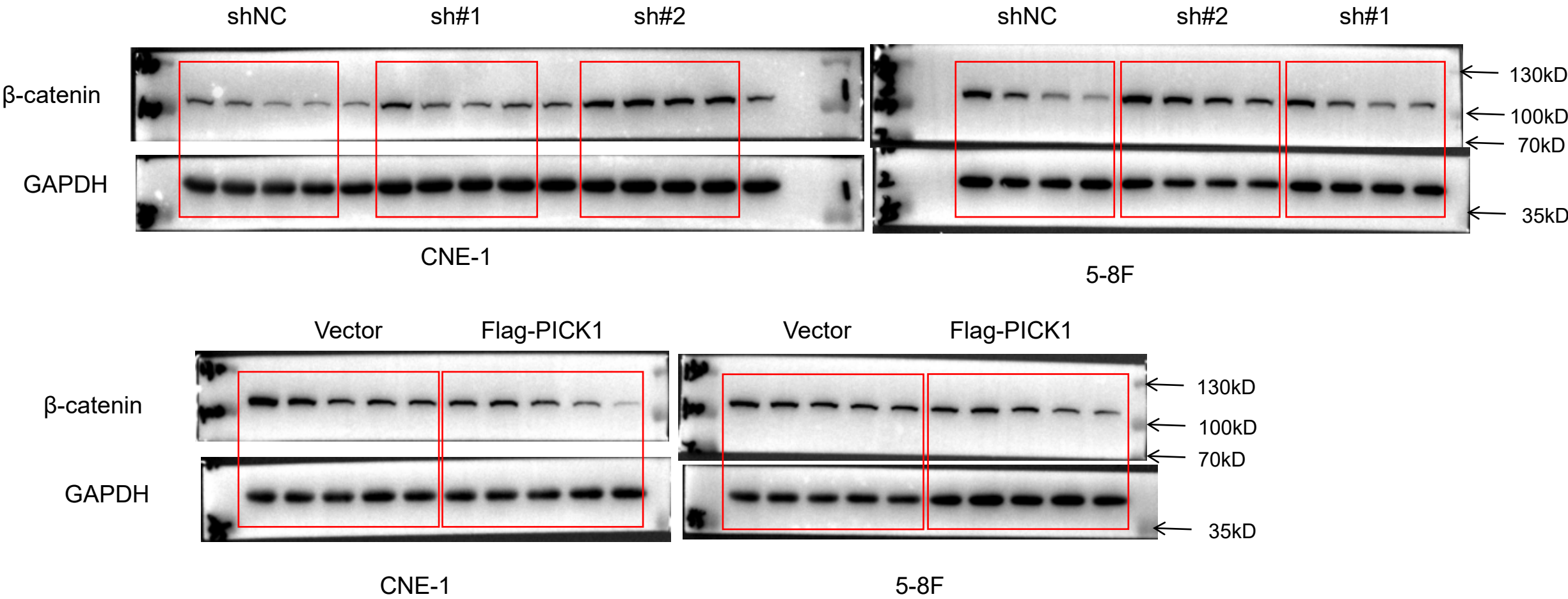

Figure 8E

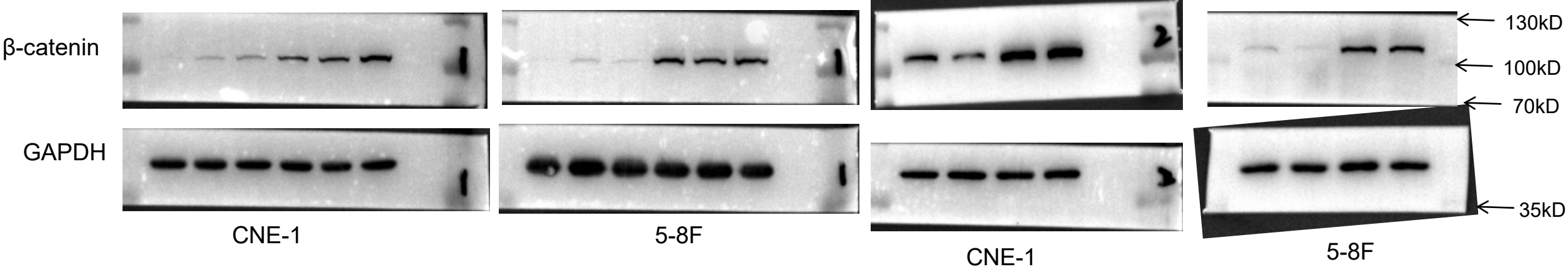

Figure 8F

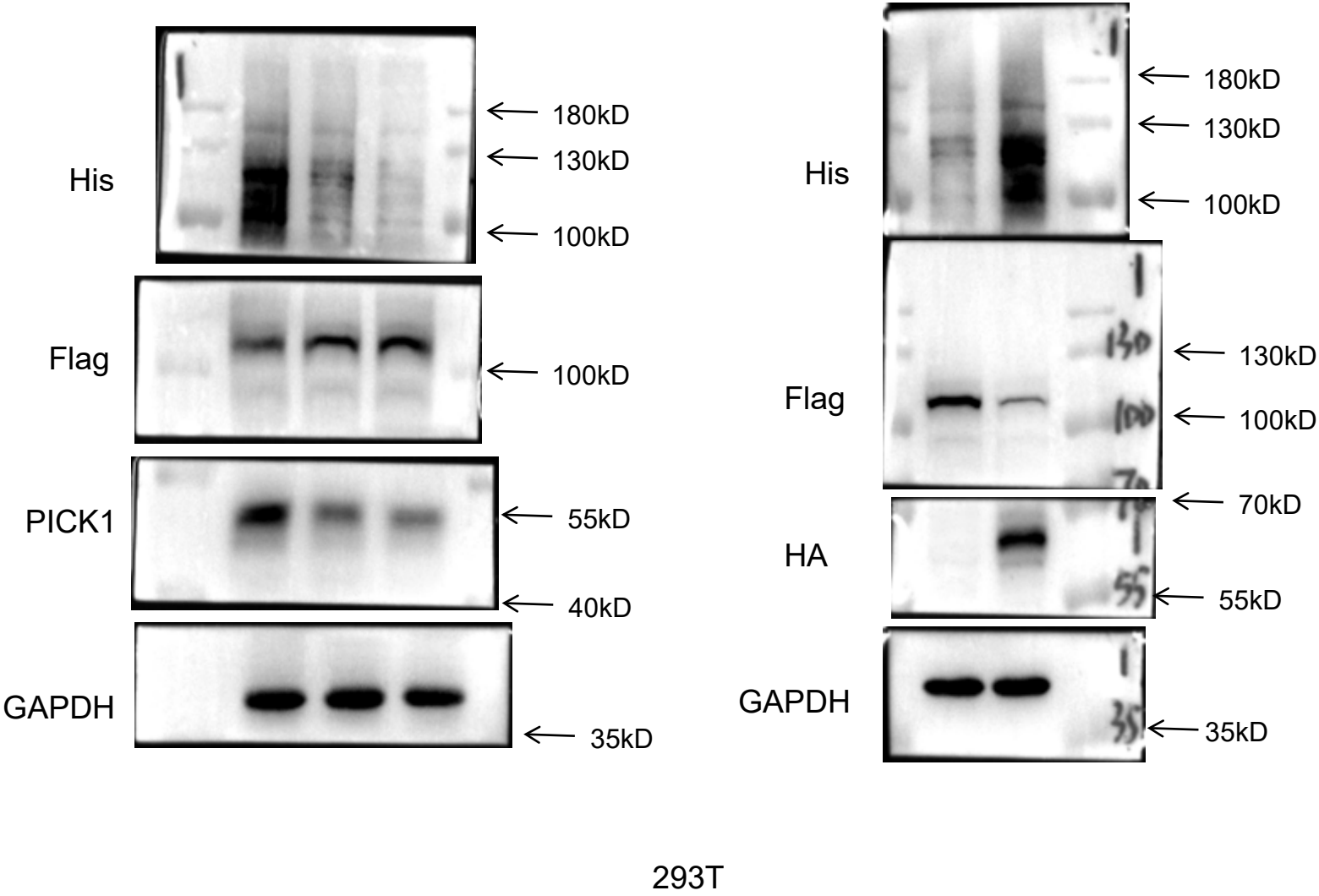

Figure 8G

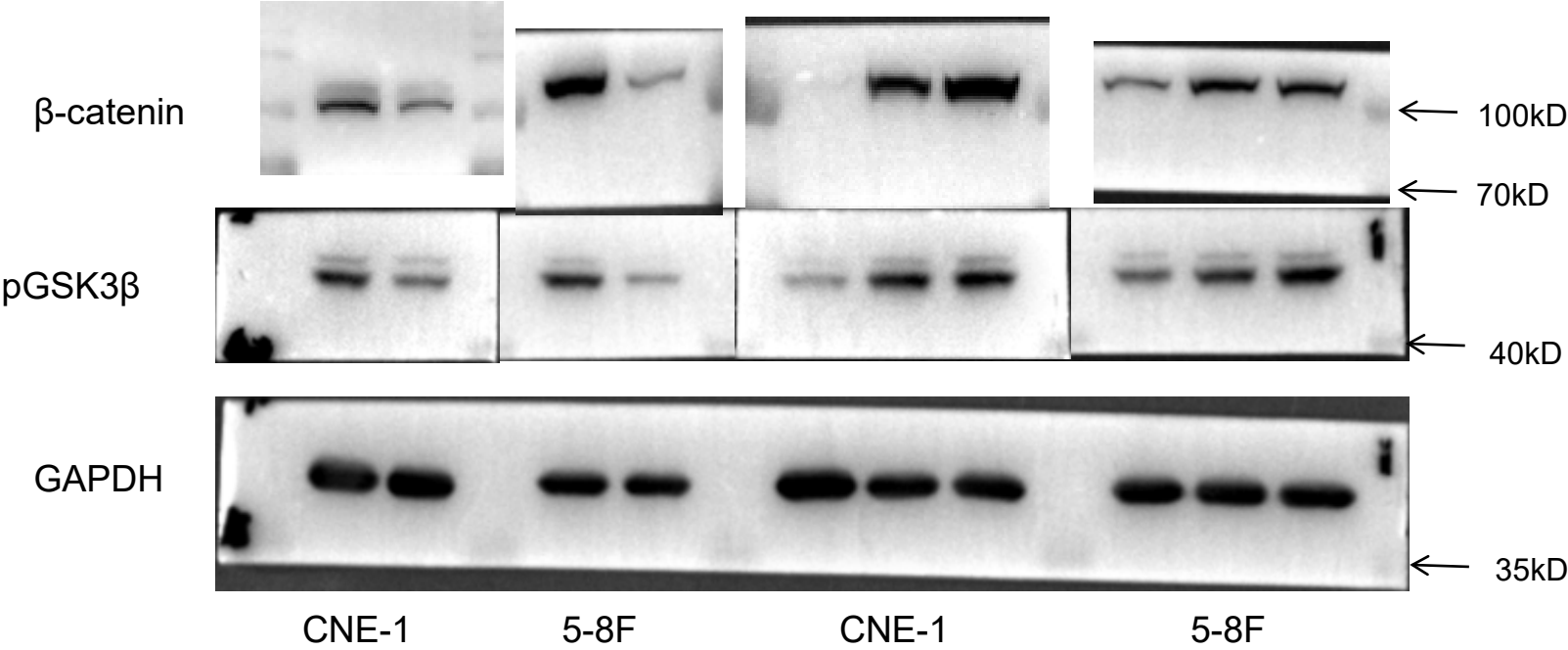

Figure 8H

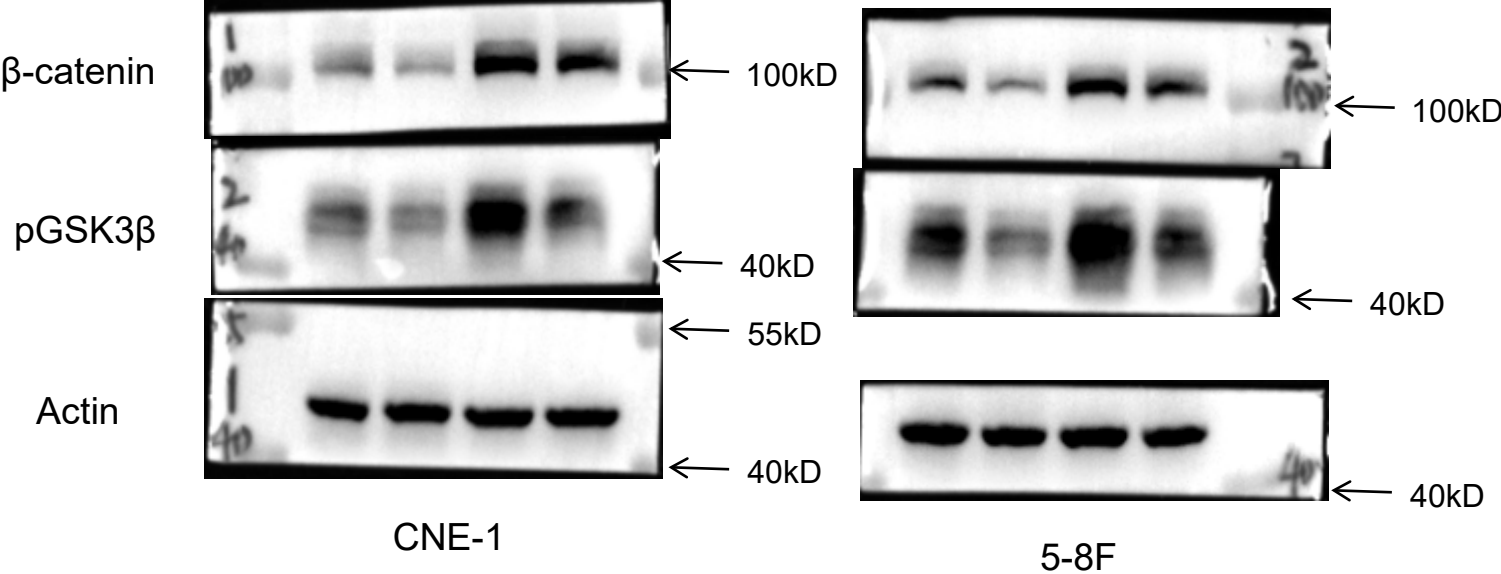

Figure S2

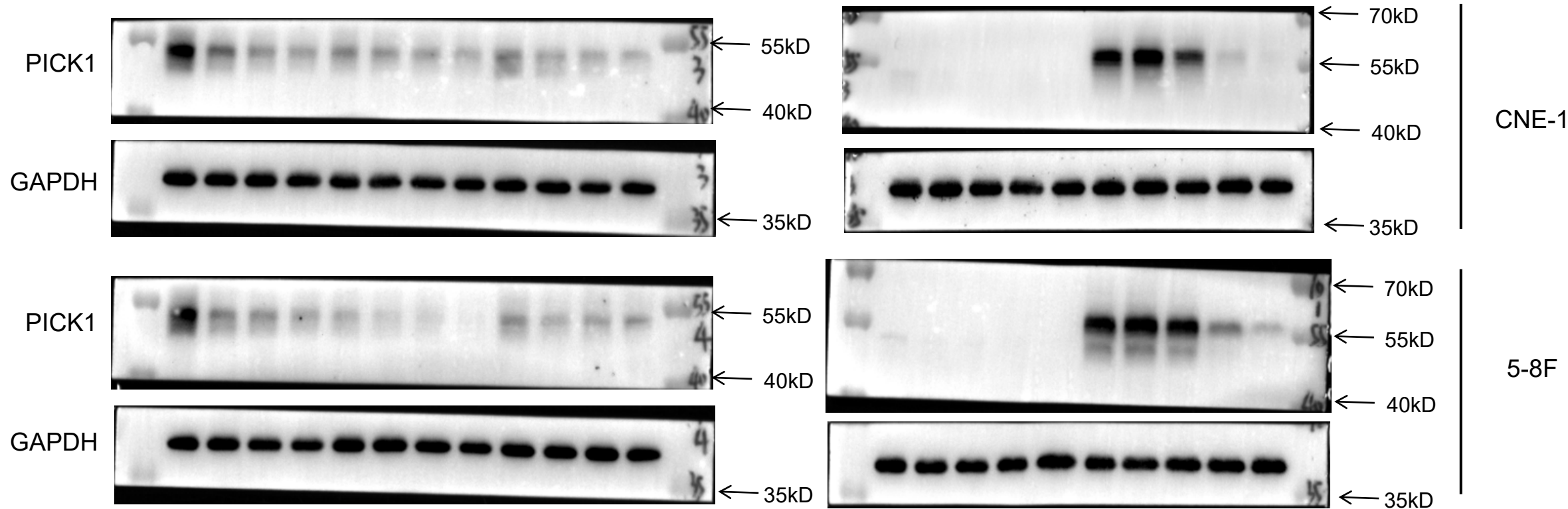

Supplement: Supplementary file 2 — Original Data File [file 41419_2024_6687_MOESM2_ESM.pdf]
